# Identification of novel biomarkers for neonatal hypoxic-ischemic encephalopathy using iTRAQ

**DOI:** 10.1186/s13052-020-00822-7

**Published:** 2020-05-24

**Authors:** Yuanyuan Zhu, Yajing Yun, Meifang Jin, Gen Li, Hong Li, Po Miao, Xin Ding, Xing Feng, Lixiao Xu, Bin Sun

**Affiliations:** 1 Department of Neonatology, Children’s Hospital of Soochow University, Suzhou, China; 2 Institute of Pediatric Research, Children’s Hospital of Soochow University, Suzhou, China

**Keywords:** Neonate, Hypoxic-ischemic encephalopathy, Isobaric tags for absolute and relative quantification, Haptoglobin, S100A8

## Abstract

**Background:**

A prompt diagnosis of HIE remains a challenge clinically. This study aimed to identify potential biomarkers of neonatal hypoxic-ischemic encephalopathy (HIE) via a novel proteomic approach, the isobaric tags for absolute and relative quantification (iTRAQ) method.

**Methods:**

Blood samples were collected from neonates with mild (*n* = 4), moderate (*n* = 4), or severe (*n* = 4) HIE who were admitted to the neonatal intensive care unit of Children’s Hospital of Soochow University between Oct 2015 and Oct 2017. iTRAQ was performed in HIE patients and healthy controls (*n* = 4). Bioinformatics analyses including Gene Ontology and KEGG pathway enrichment analysis were performed to evaluate the potential features and capabilities of the identified differentially expressed proteins.

**Results:**

A total of 51 commonly differentially expressed proteins were identified among the comparisons between mild, moderate, and severe HIE as well as healthy controls. Haptoglobin (HP) and S100A8 were most significantly up-regulated in patients with HIE and further validated via real-time PCR and western blotting. The differentially expressed proteins represented multiple biological processes, cellular components and molecular functions and were markedly enriched in complement and coagulation cascades.

**Conclusions:**

HP and S100A8 may serve as a potential biomarker for neonatal HIE and reflects the severity of HIE. The complement and coagulation cascades play crucial roles in the development of neonatal HIE.

## Introduction

Neonatal hypoxic ischemic encephalopathy (HIE) is a severe form of neonatal brain damage caused by an asphyxia-related decrease in cerebral blood flow and hypoxia. It is estimated that 4 million out of approximately 130 million newborns suffer from asphyxia worldwide per year, of which 1 million die and 1 million develop serious and long-term sequelae [1]. In China, the incidence of newborn HIE is about 54,000–108,000 in up to 20 million live births per year, of which 8000–20,000 die in the neonatal period, and 25–30% of the survivors are affected by long-term sequelae such as cerebral palsy, epilepsy, and mental retardation [2].

An early diagnosis of HIE is essential for subsequent treatment and prognosis. However, prompt diagnosis of HIE remains a challenge clinically partly due to its unclear pathogenesis and rapid progression. Previous studies revealed that biomarkers such as brain-specific proteins (neuron-specific enolase (NSE), S100B, ubiquitin carboxy-terminal hydrolase-L1, total Tau) and cytokines, including interleukin (IL)-6, IL-1β, IL-10, IL-13, interferon-gamma, tumor necrosis factor alpha and brain-derived neurotrophic factor, are helpful in the diagnosis of HIE and prediction of neurodevelopment outcomes [3–6]. However, optimal sensitivity and specificity of these biomarkers have not been achieved, which has limited their clinical application [5].

Advances in proteomics technology have brought new hope for the identification of novel biomarkers for a number of diseases. As a critical component of post-genomic research, proteomics includes two research strategies, namely “complete proteomics” and “differential proteomics”. Differential proteomics is mainly applied to screening for differences in proteomes between different samples. The proteomics diversity further reveals the physiological and pathological status of cells, the underlying mechanisms of cell activity, and the features of involved key proteins [7]. To date, to our knowledge, there has been no study evaluating biomarkers of neonatal HIE using proteomics technology.

In the current study, the similarities and differences in serum protein expression between newborn HIE patients and healthy newborns were detected by a novel isobaric tag for absolute and relative quantitation (iTRAQ)-based quantitative clinical proteomics approach. We attempted to identify novel biomarkers for neonatal HIE. Our results not only provide a theoretical basis for uncovering the disease pathogenesis of neonatal HIE, but also may serve as a scientific basis for the development of further treatment strategies.

## Methods

### Study cohort

In this prospective study, full-term newborn infants diagnosed with HIE and admitted to the neonatal intensive care unit of Soochow University Children’s Hospital between October 2015 and October 2017 were enrolled as the HIE group. The diagnosis of HIE was based on the modified 2004 HIE diagnostic and indexing criteria of the Neonatology Group of the Pediatric Society of the Chinese Medical Association [8]. All HIE patients were evaluated within the first 3 days after birth. Healthy full-term neonates born at the municipal hospital of Suzhou during the same period whose characteristics including gender, birth weight, and delivery patterns were well matched with the HIE group were selected as the controls. Patients with severe infection, malformation, bilirubin encephalopathy or incomplete data were excluded from the HIE group. Patients were excluded from the control group if they had a history of asphyxia or if the pregnant mother had underlying diseases. A total of 12 HIE patients (4 for each HIE group, mild HIE, moderate HIE and severe HIE) and 4 healthy controls were selected for iTRAQ analysis. These HIE patients were randomly selected from the 57 initially enrolled patients, which included 27 cases of mild HIE, 16 of moderate HIE, and 14 of severe HIE. Sixteen healthy patients were included as the controls.

### Data collection

Characteristics including gender, gestational age, birth weight, delivery mode, and length of hospital stay were recorded. General conditions including consciousness, muscle tone, reflexes, convulsions, respiratory failure, and pupillary changes were evaluated. Laboratory measurements and radiological imaging were performed.

### Sample preparation

All patients and healthy controls were at approximately the same age (37–38 weeks). Blood samples (3 ml) were collected before the initiation of treatment after birth. For healthy controls, identical volumes of blood samples were collected. Blood samples were not collected during therapeutic hypothermia. All blood samples were collected in EDTA tubes, and plasma was obtained by centrifugation at 3000 g for 10 min. Aliquots of plasma were taken and stored at − 80°C. Immunoglobulin (IgG) and albumin were removed from the samples (40 μl) using the Qproteome Albumin/IgG Depletion Kit (Qiagen, Valencia, CA, USA). After depletion of these high-abundance proteins, the samples were centrifuged at 12000 g. The protein content of each sample was determined using the Pierce BCA Protein Assay (Thermo Fisher Scientific, Rockford, IL, USA). Proteins (15 μg) from each sample were separated by sodium dodecyl sulfate (SDS)-polyacrylamide gel electrophoresis (PAGE) on 12% polyacrylamide gels and stained with Coomassie blue. The stained gel was scanned by the Image Scanner (GE Healthcare, USA) at a resolution of 300 dots per inch.

Extracted proteins (100 μg) were digested with trypsin at 37 °C. Peptides were eluted from filters with 100 mM ammonium bicarbonate buffer and purified. iTRAQ labeling was subsequently performed using an 8-plex iTRAQ labeling kit (AB Sciex, USA; catalogue: 4381664) according to the manufacturer’s protocol. Controls were labeled with iTRAQ reagents 113 and 114; mild HIE samples with iTRAQ reagents 115 and 116; moderate HIE samples with 117 and 118; severe HIE samples with 119 and 121. The labeled digest samples from the four groups were combined into one tube.

### LC-MS/MS analysis

An EASY-nLCTM 1200 UHPLC system (Thermo Fisher, Waltham, MA, USA) was coupled to a Orbitrap Q Exactive HF-X mass spectrometer (Thermo Fisher). Columns packed with ChromXP C18 (100 μm × 3 cm, 3 μm, 150 Å, Eksignet) were used for online trapping and desalting, while columns packed with Chrom XP C18 (75 μm × 15 cm, C18, 3 μm, 120 Å, Eksigent) were utilized for analytical separation. The mobile phase A was composed of acetonitrile/water/formic acid (ACN-H2O-FA 2: 98: 0.1, v/v/v), and the mobile phase B of ACN-H2O-FA (95: 5: 0.1, v/v/v). Samples were loaded on the column with trapping and desalting carried out at 3 μL/min for 10 min using mobile phase A. Analytical separation was performed at a flow rate of 300 nL/min. Key parameter settings included first grade MS parameters of a resolution of 70,000, Automatic Gain Control (AGC) target of 1e6, and scan range of 300 ~ 1800 m/z; and second grade MS parameters of resolution of 35,000, AGC target of 5e5, maximum Ion Accumulation Time (IT) of 100 ms, preferred dynamic exclusion time of 30s, and Normalized Collision Energy (NCE)/stepped NCE of 28.

### Database search and analysis of differentially expressed proteins

Protein identification was performed using the Proteome Discoverer™ software (version 1.3, Thermo Scientific). The RAW files were converted to a ProteinPilot compatible Mascot Generic Format (MGF) with pre-selected iTRAQ reporter ions. The files were searched against the Uniprot *Homo sapiens* database with the following search parameters: sample type, iTRAQ 8-plex (Peptide Labeled); Cys-alkylation, iodoacetamide; digestion, trypsin; instrument, Q Exactive; database, *Homo sapiens*; and Fasta.

Proteins identified at a 1% false discovery rate (FDR) with a confidence threshold of 95% (Proteinpilot Unused score ≥ 1.2) in all runs and *p* values less than 0.05 in at least one pairwise comparison were retained.

### Bioinformatics analyses of differentially expressed proteins

The functional analysis was performed through the OmicsBean cancer data analysis platform. The linked databases were Quick GO (https://www.ebi.ac.uk/QuickGO/) and Kyoto Encyclopedia of Genes and Genomes (KEGG) pathway (http://www.kegg.jp/kegg/patyway.html). Gene ontology (GO) analysis included biological processes, cellular components and molecular functions. KEGG pathway analysis was conducted to identify pathway clusters. A *p* value less than 0.05 was set as the cutoff criterion.

### Polymerase chain reaction (PCR) analysis and western blot analysis for validation of biomarkers

Total RNA from the samples was isolated in two steps using Trizol reagent (Invitrogen, Carlsbad, CA, USA) followed by RNeasy (Qiagen) purification and then subjected to reverse transcription. Haptoglobin (HP), S100A8, apolipoprotein E (APOE), and apolipoprotein M (APOM) expression levels were amplified with primers (5′-TAGAGACCGAGTGTCCTCA-3′, 5′-CGCCCATCTTTATCACCAGA-3′, 5′-CAGCACAGTCCCCGAAAAGAA-3′, 5′- CAGTCGCATACCAGGTGTCC-3′, 5′-TGACGCTGGGGCTGGCATTG-3′, 5′-GCTCTTGCTGGGGCTGGTGG-3′, 5′- GTTGCTGGTCACATTCCTGG-3′, 5′- GCAGGTAATCCCAAAAGCGAC-3′, 5′- GCTACCATCCGCATGAAAGAT-3′, 5′- CTGGCCTGTCTCATTCAGCA-3′). The PCR reactions were performed in a 10 μL volume containing a 2× SYBR Green Master Mix and the reaction was carried out using a fluorescent quantitative real-time PCR (Roche LightCycler 480, Roche, Switzerland). The amplification parameters were 95 °C for 5 min; followed by 45 cycles of 95 °C for 1 min and, 60 °C for 20 s, and 72 °C for 20 s. β-actin was used as an internal control to ensure cDNA quality and loading accuracy. For every transcript, each cDNA sample was analyzed in triplicate. The assessment of expression comparing different targets was determined by the ddCt comparative threshold (^ΔΔ^Ct) method. *P*-values were determined by a two-tailed paired Student’s t-test.

Plasma proteins were separated by SDS-PAGE and transferred to polyvinylidene fluoride (PVDF) membranes. The membranes were blocked with blocking reagent and then incubated with primary antibody at a concentration of 1: 1000 (Santa Cruz Biotechnology, Santa Cruz, CA, USA), overnight at 4 °C, followed by secondary antibody at a concentration of 1:5000 (Santa Cruz Biotechnology) for 1 h at room temperature. A Western Imaging System (Thermo Fisher Scientific) was used to obtain the images and analyze target protein expression.

### Statistical analysis

Statistical analysis was carried out using SPSS Statistics 22.0 (IBM, Armonk, NY, USA). Graphs were prepared using GraphPad Prism 7 software (GraphPad, Inc., San Diego). Variables were described as mean ± standard deviation or median (interquartile) as appropriate. Continuous variables were compared with a t-test or Mann-Whitney U test, whereas categorical data were compared with chi-square test or Fisher’s exact test. Differences in protein expression between groups were analyzed using a t-test. Values of p less than 0.05 were considered statistically significant.

## Results

### Baseline characteristics of HIE patients and healthy controls

Of the 12 cases of HIE for which iTRAQ analysis was performed, male and female patients accounted for 7 (58.3%) and 5 (41.7%) cases, respectively. Blood samples were withdrawn before the initiation of treatment within a mean time of 5.19 ± 2.12 h after birth. For healthy controls, identical volumes of blood samples were withdrawn at a mean time of 6.46 ± 1.79 h after birth. There were no significant differences in gender and time before sampling between the HIE and control groups (both *p* > 0.05).

### Differentially expressed proteins identified by iTRAQ analysis

**Fig. 1:**
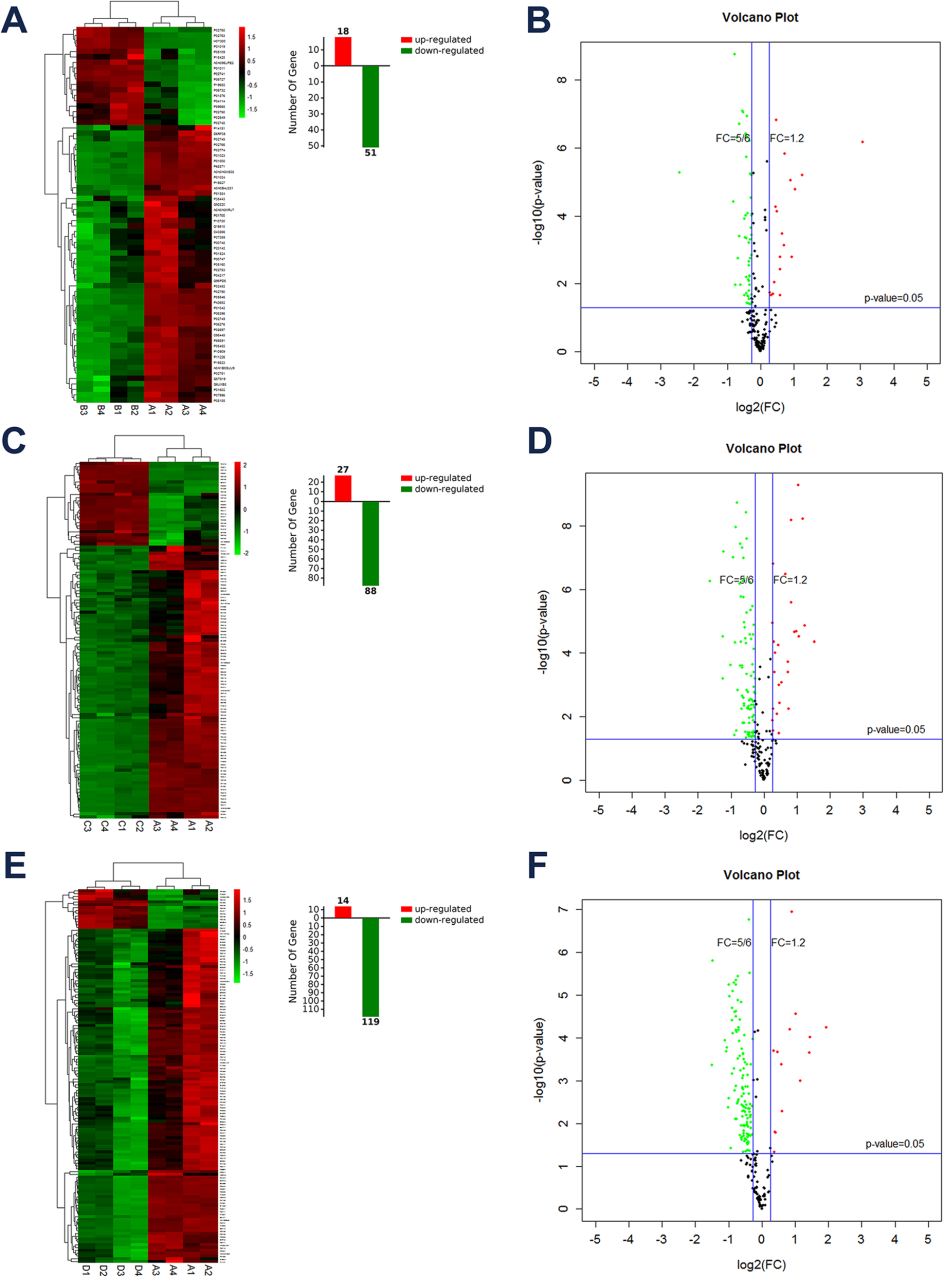
Differentially expressed proteins among HIE patients and healthy controls. **a**, **b** Clustering heatmap and Volcano map of proteins differentially expressed between mild HIE and controls. **c**, **d** Clustering heatmap and Volcano map for the comparison of moderate HIE vs control. **e**, **f** Clustering heatmap and Volcano map for the comparison of severe HIE vs control. The green dots indicate down-regulated proteins, and the red dots indicate up-regulated proteins. The black dots depict the non-significant differentially expressed proteins

From the comparison of mild HIE cases versus controls, 69 differentially distributed proteins were detected, including 18 up-regulated proteins and 51 down-regulated proteins. The significantly differentially expressed proteins were illustrated in a volcano plot, and the expression levels of proteins in each sample were visualized in a hierarchical clustering heatmap (Fig. 1a, b). From the comparison of moderate HIE cases versus controls, 115 differentially expressed proteins were detected, of which 27 were up-regulated and 88 were down-regulated (Fig. 1c, d). From the comparison of severe HIE cases versus controls, 133 differentially expressed proteins were identified, including 14 up-regulated proteins and 119 down-regulated proteins (Fig. 1e, f).

**Fig. 2:**
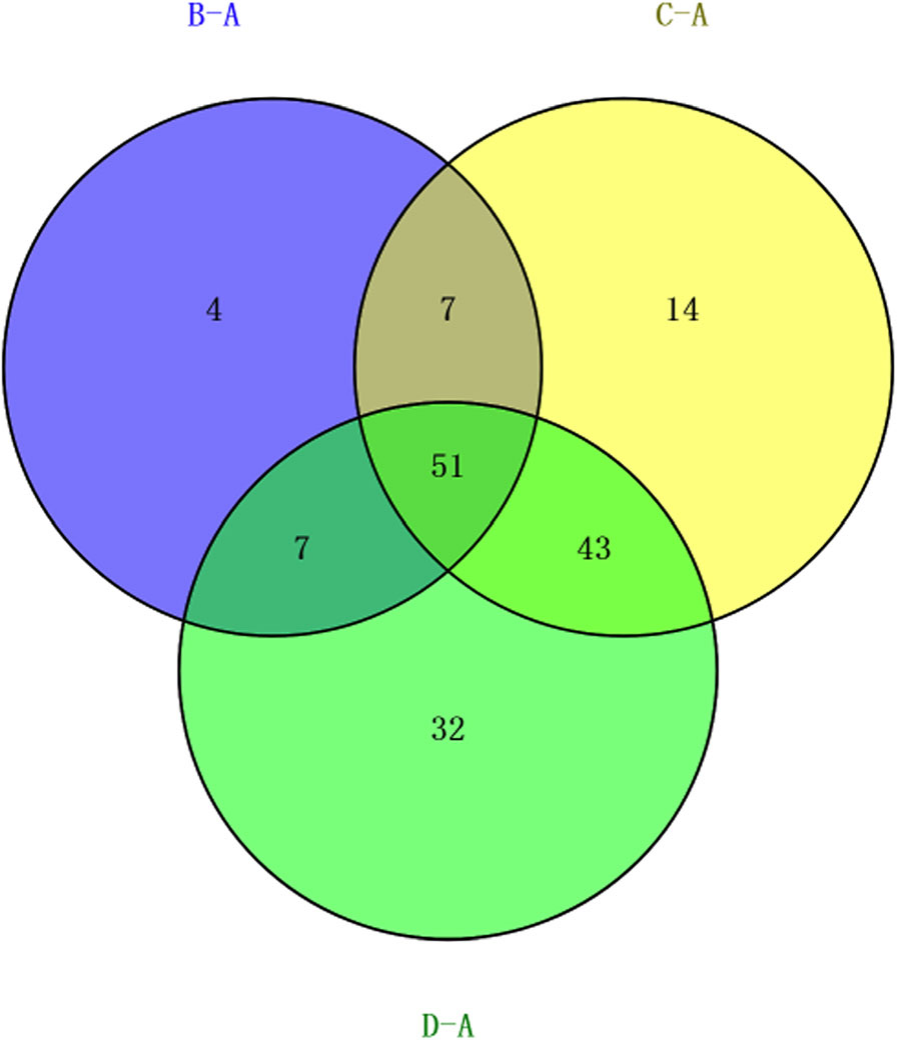
Venn diagram depicting 51 commonly differentially expressed proteins in all three comparisons of mild HIE vs controls, moderate HIE vs controls, and severe HIE vs controls

To identify commonly differentially expressed proteins within the above three comparisons, a Venn diagram was drawn and showed that 51 proteins were identified in multiple comparisons (Fig. 2, Table 1).

**Table 1:** List of the most differentially expressed proteins in comparisons of mild HIE vs controls, moderate HIE vs controls, and severe HIE vs controls

Description	Mild HIE vs controls (fold change)	*p*	Moderate HIE vs controls (fold change)	*p*	Severe HIE vs controls (fold change)	*p*
S100A8	1.92	0.002	2.87	< 0.001	3.85	< 0.001
C-reactive protein	2.38	< 0.001	2.35	< 0.001	2.69	< 0.001
Apolipoprotein E	1.21	0.019	0.73	0.003	0.72	0.004
Complement C1q subcomponent subunit A	0.83	0.001	0.78	< 0.001	0.70	< 0.001
Coagulation factor V	0.75	0.019	0.72	0.006	0.68	0.004
Vitamin D-binding protein	0.81	0.002	0.69	< 0.001	0.67	< 0.001
Coagulation factor XII	0.72	0.022	0.63	0.002	0.54	0.001
Apolipoprotein M	0.83	< 0.001	0.60	< 0.001	0.50	< 0.001
Platelet factor 4 variant	0.59	0.011	0.42	0.001	0.35	< 0.001
Haptoglobin	8.44	< 0.001	1.38	0.033	1.24	0.080
S100A9	1.37	0.085	1.68	0.006	2.22	< 0.001

### Bioinformatics analyses of differentially expressed proteins

**Fig. 3:**
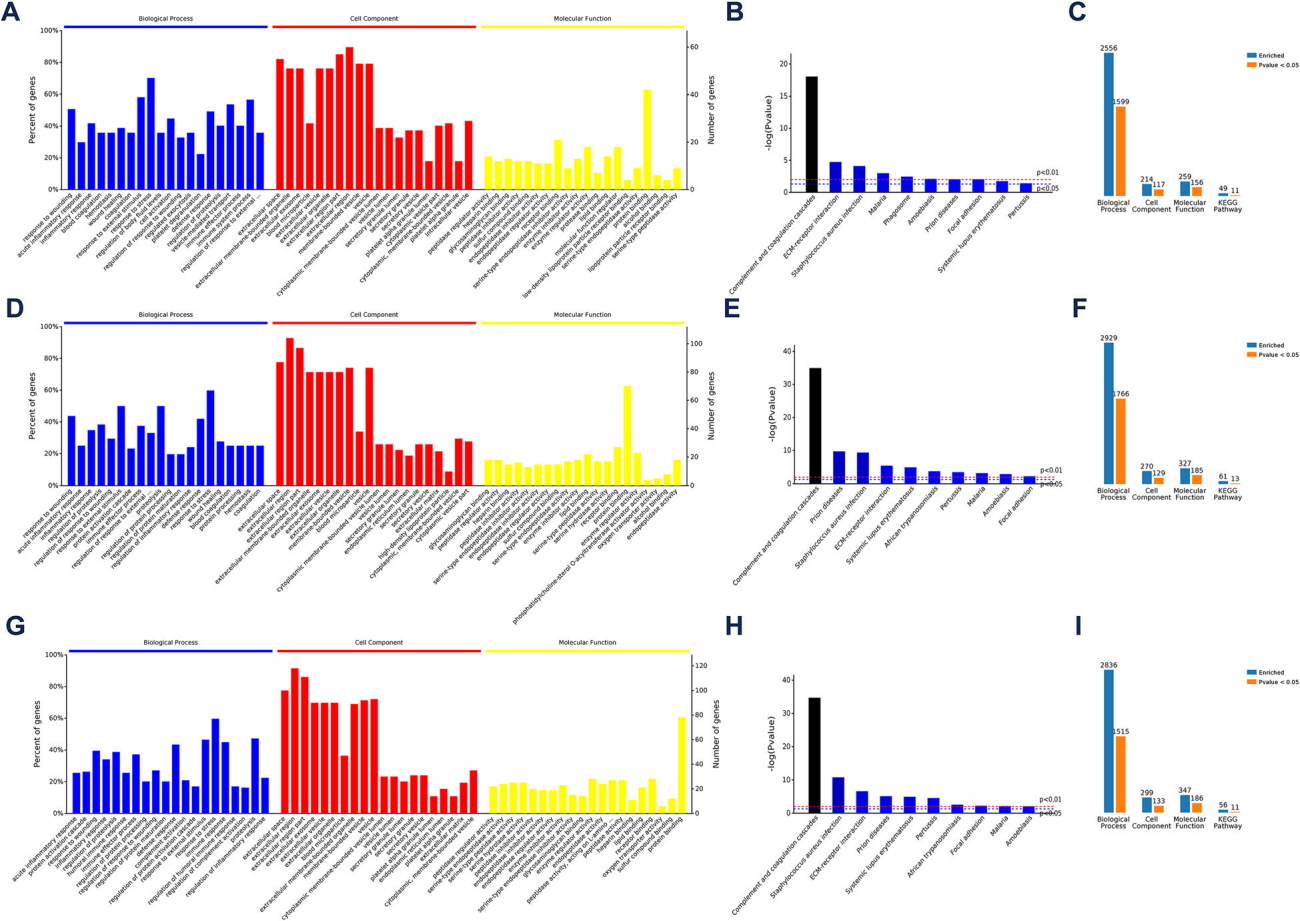
Overview of GO enrichment analysis and KEGG pathway analysis. **a** Differentially expressed proteins (mild HIE vs controls) were associated with major biological processes (BPs), cell localizations (CCs) and molecular functions (MFs). **b** Major pathways involved (mild HIE vs controls). **c** Summary of GO enrichment analysis and KEGG pathway analysis for mild HIE vs controls. **d**, **e**, **f** Features and participating pathways for differentially expressed proteins between moderate HIE vs controls. **g**, **h**, **i** Features and participating pathways of differentially expressed proteins between severe HIE vs controls

GO analysis explored the biological processes (BPs), cell components (CC) and molecular functions (MF) associated with the top 20 most positively and negatively regulated proteins. Comparing mild HIE cases with controls, the BP involvement of the most differentially expressed proteins included the injury response, acute inflammatory response, and inflammatory response. Involved CCs were extracellular space, extracellular membrane organelles, and exosomes. Participating MFs included peptidase regulation agent, heparin binding, and glycosaminoglycan binding. KEGG analysis showed that differentially expression proteins were markedly enriched in complement and coagulation cascades. The proteins most differentiated between moderate HIE cases and controls were associated with BPs, CCs and MFs similar to those of the proteins differentially expressed between mild HIE cases and controls. Slight differences in the BPs, CCs and MFs were detected (Fig. 3).

### Validation of the identified potential biomarkers

**Fig. 4:**
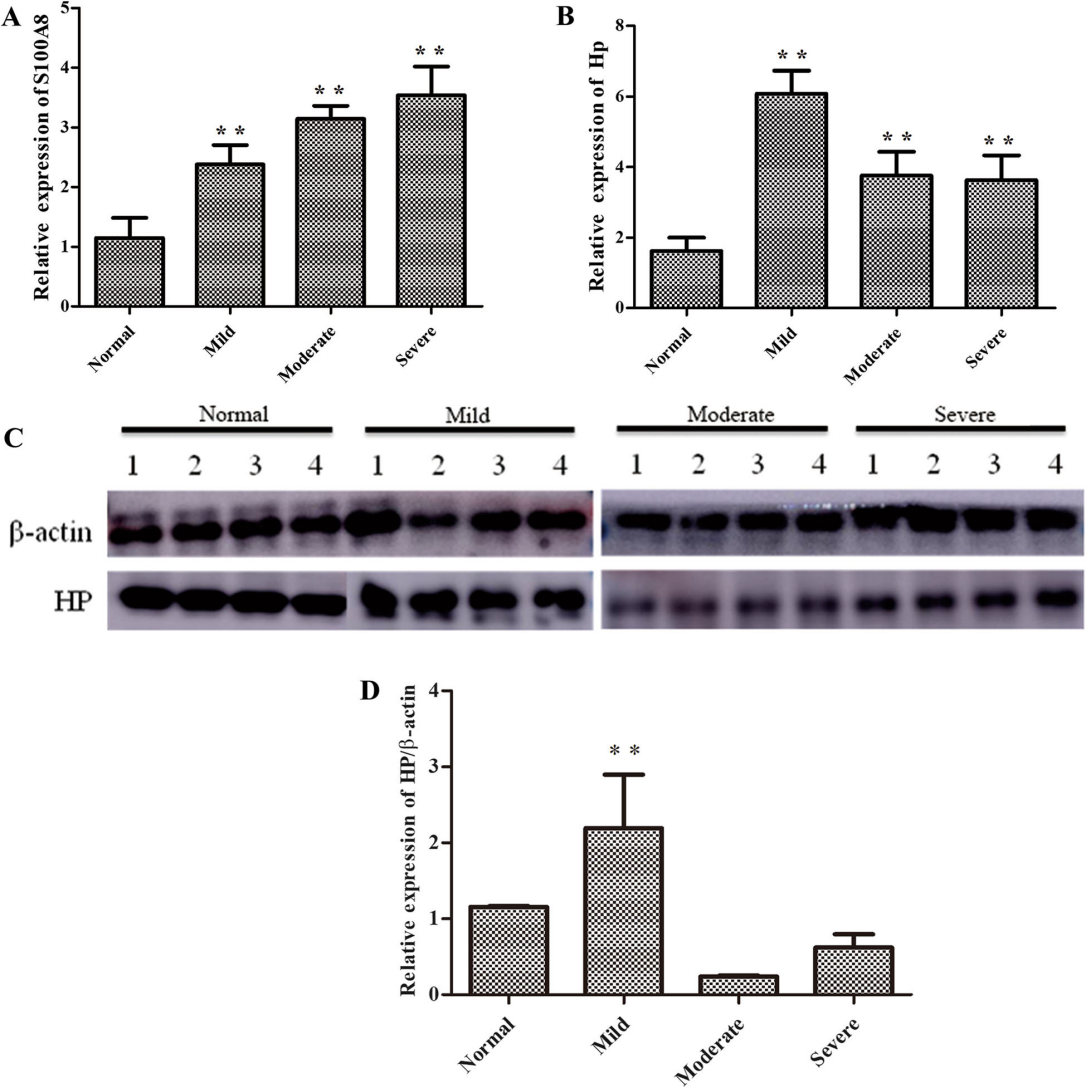
**q**PCR and Western Blot analysis of HP and S100A8 expression. **a**, **b** Relative expression of S100A8 and HP in qPCR. **c**, **d** Relative expression of HP in Western Blot

Eight healthy newborns, 19 patients with mild HIE, 8 with moderate HIE, and 6 with severe HIE were chosen for further validation of the iTRAQ findings. The PCR results showed that, consistent with the findings of iTRAQ, HP and S100A8 were up-regulated in patients with HIE. However, conflicting results for the proteins APOE and APOM were observed. Western blot analysis (*n* = 4 for each group) further verified the increased expression of HP and S100A8 in patients with HIE (Fig. 4).

## Discussion

The current study investigated diagnostic biomarkers of neonatal HIE by screening differentially expressed proteins between HIE patients and healthy controls. To the best of our knowledge, this study was the first to detect potential biomarkers of neonatal HIE using novel iTRAQ technology. We found that S100A8 may serve as a potential biomarker and HP was closely linked with HIE. Bioinformatics analysis suggested that the complement and coagulation cascades play critical roles in the development of neonatal HIE.

Our results confirmed that S100A8 expression was significantly elevated in all three groups of HIE patients and reflected the severity of HIE. No evidence of the relationship between S110A8 and HIE has been previously reported. However, as an important S100 protein family member, S100A8 is involved in a number of physiological functions including the inflammatory response and calcium homeostasis regulation through combination with S100A9 [9]. The S100A8/A9 complex has been reported to be capable of inhibiting excessive oxidation and facilitating production of pro-inflammatory cytokines [10, 11]. The S100A8/A9 complex also has been shown to participate in multiple diseases such as asthma and rheumatoid arthritis [12, 13]. For patients with brain injury, the S100A8/A9 complex was shown to be involved in the early stage of cerebral ischemia-reperfusion injury [14]. Previous studies demonstrated that S100A8/A9 mediates early inflammatory responses to neuronal damage by activating microglia Toll-like receptor 4 (TLR4)/nuclear factor kappa B (NF-κB) and receptor for advanced glycation endproducts (RAGE)/NF-κB signaling pathways [15]. Therefore, S100A8 may be a potential biomarker for the diagnosis of HIE and also the monitoring of the disease progression.

The HP protein inhibits the hemoglobin-related cerebral inflammatory response, and elevated HP expression may indicate the development of HIE [16]. We observed a consistent increase in HP expression in patients independent of the severity of HIE, and the trend in expression was not parallel with the severity. HP is an acute phase protein that is the most abundant endogenous hemoglobin-binding protein in plasma [17]. Studies have shown that HP is expressed in the brain and has a neuroprotective effect [18]. HP can protect neurons from the cytotoxic effects of hemolysates after intracranial hemorrhage, which is one of the major manifestations of HIE. HP acting as an antioxidant could remove free hemoglobin from circulation and protect subsequent impairment due to oxygen free radicals [16]. In patients with severe spinal cord injury, HP expression is increased [19]. Previous in vivo and in vitro studies reported that up-regulation of HP in brain is mainly mediated by oligodendrocytes [20]. However, HP expression has rarely been investigated in newborns previously. Our findings imply that HP could be a definitive marker of HIE, especially mild HIE or early-stage of HIE.

Bioinformatics analysis suggested that the injury response, inflammatory response, and protein activation cascade are important biological processes during the development of HIE. More importantly, KEGG pathway analysis revealed that the complement and coagulation cascades participate the most in all groups. The complement proteins are largely present in human blood and tissue fluid as well as on the cell surface. They function in immunomodulation, immune defense and phagocytosis enhancement [21]. Studies have shown that the blood–brain barrier is impaired via activation of complement proteins, which facilitate histamine and lysosomal enzyme release, and increased vascular permeability [22]. Degeneration and necrosis of the brain induce inflammation via activating the complement system [23]. In a rat model of HIE, researchers found that receptors for C3a and C5a were predominantly expressed on microglia after HIE, and hypothermia may improve outcomes by modulating the complement system [24].

Our study based on iTRAQ demonstrated that the coagulation pathway was greatly involved in the development of HIE. Coagulopathy has been proven to be one of the major consequences of neonatal asphyxia [25, 26]. Coagulation disorders following neonatal HIE have multifactorial mechanisms. The synthesis of platelets and clotting factors are inhibited due to insufficient blood and oxygen supply [25–28]. Disseminated intravascular coagulation is frequently present in newborns who suffer from asphyxia [29, 30]. Furthermore, therapeutic hypothermia can potentially disturb hemostasis by slowing the enzymatic function of the coagulation cascade, suppressing thrombin generation, and triggering disseminated intravascular coagulation [31, 32]. Early intervention with coagulation function can improve the prognosis in neonates with HIE. Our results confirmed the concept that the coagulation pathway plays an important role in the progression of HIE.

There are several limitations in the current study. First, the study sample was small due to the limitations of iTRAQ measurement. Second, we did not validate all differentially expressed proteins. However, our findings were partly consistent with the concept that multi-organ and response systems including the oxidative/inflammatory and coagulation cascades are involved in the development of HIE [5]. The differing results among studies may be explained by differences in measurement timing and the related short-or long-term outcomes. In addition, the sensitivity and specificity of different measurements may affect the outcomes as well. Third, we did not analyze the relationship between potential biomarkers and other objective measurements reflecting long-term outcome such as brain MRI. More precise brain imaging and cerebral function assessment may provide further information for the detection of biomarkers. In previous studies assessing the correlations between biomarkers and HIE outcomes, elevated plasma brain-specific proteins including S100B in the first 24 h after birth were associated with brain injury detected by MRI in HIE newborns [4]. However, S100B was not found to be related to neurodevelopmental outcomes [4]. Predictive or prognostic biomarkers that could guide therapeutic strategies may be of critical importance.

## Conclusions

Our study based on iTRAQ identified S100A8 and HP protein as a potential biomarker for neonatal HIE and the complement and coagulation cascades as important in the development of neonatal HIE.

## Data Availability

The datasets used and/or analyzed during the current study are available from the corresponding author on reasonable request.

## Abbreviations

HIE: Neonatal hypoxic ischemic encephalopathy
NSE: Neuron-specific enolase
IL-6: Interleukin-6
iTRAQ: Isobaric tag for absolute and relative quantitation
IgG: Immunoglobulin
SDS: Sodium dodecyl sulfate
PAGE: Polyacrylamide gel electrophoresis
ACN-H2O-FA: Acetonitrile/water/formic acid
AGC: Automatic gain control
IT: Ion accumulation time
NCE: Normalized collision energy
MGF: Mascot generic format
FDR: False discovery rate
KEGG: Kyoto Encyclopedia of Genes and Genomes
GO: Gene ontology
PCR: Polymerase chain reaction
HP: Haptoglobin
APOE: Apolipoprotein E
APOM: Apolipoprotein M
PVDF: Polyvinylidene fluoride
